# Dielectric and Carrier Transport Properties of Silicone Rubber Degraded by Gamma Irradiation

**DOI:** 10.3390/polym9100533

**Published:** 2017-10-20

**Authors:** Daomin Min, Chenyu Yan, Yin Huang, Shengtao Li, Yoshimichi Ohki

**Affiliations:** 1 State Key Laboratory of Electrical Insulation and Power Equipment, Xi’an Jiaotong University, Xi’an 710049, Shaanxi, China; leo-chenyu.yan@stu.xjtu.edu.cn (C.Y.); huangxingyin@stu.xjtu.edu.cn (Y.H.); sli@mail.xjtu.edu.cn (S.L.); yohki@waseda.jp (Y.O.); 2 Research Institute for Materials Science and Technology, Waseda University, Shinjuku, Tokyo 169-8555, Japan; 3 Department of Electrical Engineering and Bioscience, Waseda University, Shinjuku, Tokyo 169-8555, Japan

**Keywords:** conductivity, dielectric property, gamma irradiation, SiR, trap distribution

## Abstract

Silicone rubber (SiR) is used as an insulating material for cables installed in a nuclear power plant. Gamma rays irradiated SiR sheets for various periods at temperatures of 145 and 185 °C, and the resultant changes were analyzed by examining complex permittivity spectra and surface potential decay characteristics. Three different processes, namely, instantaneous polarization, electrode polarization due to the accumulation of ions to form double charge layers at dielectric/electrode interfaces, and DC conduction caused by directional hopping of ions, contribute to the complex permittivity. By fitting the spectra to theoretical equations, we can obtain the dielectric constant at high frequencies, concentration and diffusion coefficient of ions and DC conductivity for the pristine and degraded samples. The instantaneous polarization becomes active with an increase of dose and ageing temperature. The thermal expansion coefficient estimated from the temperature dependence of dielectric constant at high frequencies becomes smaller with an increase in dose, which is in good agreement with the experimental results of the swelling ratio. Additionally, trap distributions are calculated from surface potential decay measurements and analyzed to explain the variation in conductivity. Trap energy increases firstly, and then decreases with an increase in dose, leading to a similar change in DC conductivity. It is concluded that generations of both oxidative products and mobile ions, as well as the occurrence of chain scission and crosslinking are simultaneously induced by gamma rays.

## 1. Introduction

Silicone rubber (SiR) has excellent thermal stability, high resistance to oxidative degradation, and good electrical properties [1,2]. It has been widely used in industry as well as in our daily life [2,3]. For nuclear power plants, SiR is an important electrical insulating material for cables [2]. Energetic radiations such as gamma rays could degrade SiR, affecting its performance as an electrical insulator [2].

Gamma rays are high-energy photons and can transfer their energy to materials through photoelectric effects, including the generation of electron-hole pairs and Compton scattering [4]. When an organic polymer absorbs the energy of gamma rays, free radicals are generated [1,5,6]. In the presence of oxygen, these radicals react with oxygen to form peroxy radicals and hydroperoxides [5,6]. Then, peroxy radicals react with polymer molecules or recombine with each other, while hydroperoxides may decompose to generate inactive products. As a result, crosslinking and chain scission appear in the polymer [1,2,5,6]. These chemical reactions in polymers induced by heat and gamma irradiation cause changes in chemical composition, glass transition and melting temperatures, crystallinity, chemiluminescence, elongation at break, indenter modulus, dielectric constant, and dielectric loss, and so on [6,7,8,9,10,11,12].

Gamma irradiation induced degradation in polymers depends on dose or irradiation time, dose rate, temperature, and oxygen concentration, and so on [2,13,14,15]. With an increase in dose, the degradation of polymers becomes more severe [7,12,16]. Dose rate determines how fast free radicals are generated. Generally, polymers degrade faster at relatively higher dose rates. At a high dose rate, the reaction between free radicals and oxygen inside the material may be limited by the diffusion of oxygen, leading to non-uniform degradation [13]. In addition, high oxygen concentration can increase the rate of oxidative reactions, which will speed up the ageing process [13]. Since chemical reactions are faster at relatively higher temperatures, the radiation-induced degradation of polymers will be accelerated with an increase in temperature [2,14].

Monitoring the condition of polymeric insulating materials and clarifying the degradation mechanism is important for determining the remaining life of power cables. Dielectric spectroscopy has been used as a non-destructive tool to investigate the degradation of polymers [11,12,16,17]. Both dielectric constant and dielectric loss factor change monotonically with elongation at break, indicating that they can used as indicators of cable degradation [11,12]. Dielectric spectroscopy can also be used to estimate the concentration of ions and the ionic diffusion coefficient [18,19], providing details to explicate the degradation mechanism. In the present work, the dielectric spectra of pristine and aged SiR samples were measured and the spectra obtained were analyzed to examine the effects of ageing on the above-mentioned parameters.

## 2. Experimental Procedure

The samples were crosslinked SiR sheets in the shape of a square, with each side length of about 15 cm and a thickness of about 0.55 mm, which were supplied by a Japanese nuclear power cable manufacturing company (Tokyo, Japan). The material was reinforced by talc, and its density was measured to be about 1.20 g⋅cm^−3^. The average molecular weight between crosslinks of pristine SiR was estimated to be about 2.25 × 10^3^ g⋅mol^−1^ by Flory-Rehner model [1,13] based on solvent swelling experiments [7]. The spectrum observed by Fourier-transform infrared spectroscopy (Shimadzu Corporation, Kyoto, Japan) is similar to that of polydimethysiloxane [7]. The gamma irradiation was conducted for 900 and 1500 h using a ^60^Co gamma ray source with a dose rate of 150 Gyh^−1^ at temperatures *T*_ir_ of 145 and 185 °C in order to accelerate the ageing process. By adding the unirradiated sample to the above four samples, we used five samples in total.

Aluminum electrodes with a diameter of 20 mm were pasted on both sides of the sample with silicone oil similarly to our previous papers [12,20,21]. Then the sample was put in a vacuum chamber with a pressure below 5 Pa. Complex permittivity was measured by applying an ac voltage of 3 V_rms_ in a frequency range from 10^−2^ or 10^−3^ to 10^4^ Hz at various temperatures from 100 to 200 °C using an impedance analyzer (126096, Solartron, Farnborough, UK). In addition, surface potential decay experiments were conducted by needle-plate electrode system with needle electrode and plate electrode voltages of −12 and −9 kV, at a temperature of 50 °C. Samples were charged for about 2 min, then, their surface potentials were measured by a non-contact potential probe (Trek P0865, Trek Inc., Lockport, NY, USA).

## 3. Analysis of Experimental Results

**Figure 1:**
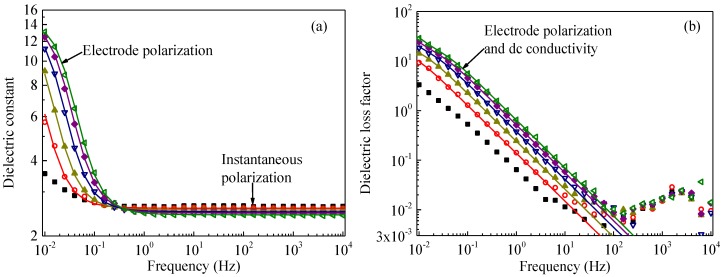
Dielectric constant *ε*_r_′ (**a**) and dielectric loss factor *ε*_r_" (**b**) of unirradiated SiR, each as a function of frequency measured at various temperatures. Symbols and solid curves represent the data obtained experimentally and numerically, respectively. The measurements were conducted at 100 °C (■), 120 °C (◯), 140 °C (▲), 160 °C (▽), 180 °C (♦), and 200 °C (◁).

**Figure 2:**
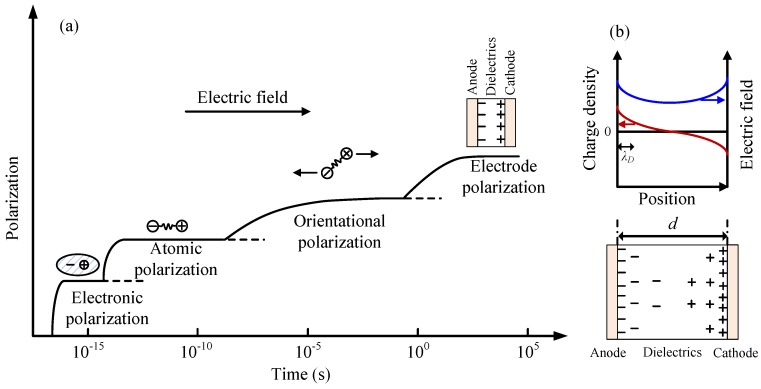
(**a**) Different types of polarization in a dielectric material under an electric field. With the increase in time, electronic, atomic, orientational, and electrode polarizations can be observed in the dielectric material [22]. (**b**) Distribution of hetero space charges accumulated in a dielectric material, which can form electrode polarization [24].

Figure 1 shows dielectric constant *ε*_r_′ and dielectric loss factor *ε*_r_′′ as a function of frequency *f* measured for pristine SiR samples at temperatures ranging from 100 to 200 °C. At frequencies above about 1 Hz, *ε*_r_′ scarcely depends on *f*. Therefore, the value of *ε*_r_′ in this frequency range can be regarded as the dielectric constant at high frequency limit *ε*_r*∞*_. The typical response time of electronic polarization is around 10^−16^–10^−15^ s in polymers, while that of atomic polarization or skeletal vibrations in polymeric molecules such as bending, twisting, expansion and contraction, or intermolecular vibration is around 10^−13^–10^−12^ s, as shown in Figure 2a [22]. Therefore, electronic polarization and vibrations of segmental chains should contribute to *ε*_r∞_ dominantly, since they are fast enough to respond to the frequency of the applied electric field. On the other hand, *ε*_r∞_ decreases with an increase in temperature. This phenomenon can be caused by thermal expansion, resulting in a decrease in the amount of atoms and molecular chains per unit volume [12,23].

The dielectric constant *ε*_r_′ at frequencies below about 1 Hz increases dramatically with a decrease in frequency and with an increase in the temperature, as demonstrated in Figure 1a. This is caused by accumulation of hetero space charges, as demonstrated in Figure 2b. If ions accumulate near an electrode, charges with the opposite polarity are induced on the electrode, which enlarges the apparent value of dielectric constant. This phenomenon is called electrode polarization [18,19,25], which has been observed in many polymers such as polyimide [26], ethylene-propylene-diene copolymer (EPDM) [12], and epoxy resin [27]. Therefore, the dielectric constant at low frequencies can be fitted to an equation of electrode polarization. Additionally, the directional migration of ions can form conduction current [28], causing the dramatic increase in the dielectric loss factor with a decrease in frequency. This phenomenon becomes obvious in a frequency range below 10^2^ Hz, as shown in Figure 1b.

By solving equations of charge conduction, charge diffusion, and charge continuity, as well as Poisson’s equation, namely by assuming the Poisson-Nernst-Planck model, Macdonald derived a formula of complex relative permittivity *ε*_r_* of electrode polarization for blocking electrodes [18,24]. The formula derived in [24] is:

(1) $$\varepsilon_{r}^{*}\left(\omega \right)=\varepsilon_{r}^{\prime }\left(\omega \right)-i\varepsilon_{r}^{\prime \prime }\left(\omega \right)=\frac{1}{2}\frac{d\varepsilon_{r\infty }\xi^{2}}{\lambda_{D}^{-2}\xi^{-1}\tanh\left(\xi d/2\right)+i\left(\omega d/2D\right)}$$

where *ω* is the angular frequency (*ω* = 2π*f*) in rad⋅s^−1^ and *d* is the sample thickness in m, while *ξ* is a frequency-dependent variable in m^−1^ and *λ*_D_ is the Debye length in m. Furthermore, *D* is the diffusion coefficient of mobile ions in m^2^∙s^−1^.

As shown in Figure 2b, the Debye length *λ*_D_ is a function of the concentration *n* of mobile ions in m^−3^ and temperature *T* in *K*, and is written as [18,19,25]:

(2) $$\lambda_{D}=\sqrt{\varepsilon_{0}\varepsilon_{r\infty }k_{B}T/\left(2nq^{2}\right)}$$

Here, *ε*_0_ is the permittivity of vacuum in Fm^−1^ and *k_B_* is the Boltzmann constant, while *q* is the charge of ions in C.

The variable *ξ* is defined by [18]:

(3) $$\xi =\sqrt{1/\lambda_{D}^{2}+i\omega /D}$$

By solving Equation (1) analytically, *ε*_r_^*^(*ω*) can be simplified to the following the Debye equation [18]:

(4) $$\varepsilon_{r}^{*}\left(\omega \right)=\varepsilon_{r\infty }+\frac{\frac{\varepsilon_{r\infty }d}{2\lambda_{D}\tanh\left(d/2\lambda_{D}\right)}-\varepsilon_{r\infty }}{1+i\omega \lambda_{D}d/2D}$$

When a certain electrode polarization exhibits a distribution of relaxation time, a Cole-Cole equation can be used to model the polarization. In addition, the contribution of DC conductivity should be considered in analyzing the behavior of *ε*_r_^*^(*ω*) of a dielectric material. Regarding this, *ε*_r_^*^(*ω*) can be expressed as follows [18,19,29,30]:

(5) $$\varepsilon_{r}^{*}\left(\omega \right)=\varepsilon_{r\infty }+\frac{\frac{\varepsilon_{r\infty }d}{2\lambda_{D}\tanh\left(d/2\lambda_{D}\right)}-\varepsilon_{r\infty }}{1+{\left(i\omega \lambda_{D}d/2D\right)}^{1-\gamma }}+\frac{\sigma_{\mathrm{DC}}}{i\varepsilon_{0}\omega }$$

Here, the second term is a Cole-Cole type electrode polarization and the third term represents the loss caused by DC conductivity *σ*_DC_ in S·m^−1^, while *γ* is a parameter representing the distribution of relaxation time.

Equation (5) depends on *D*, *n*, *σ*_DC_, and *γ.* If the frequency spectra of *ε*_r_^*^(*ω*) obtained experimentally for pristine and aged samples were fitted to Equation (5) numerically by the least squares method, the values of *D*, *n*, *σ*_DC_, and *γ* at various temperatures can be obtained.

**Figure 3:**
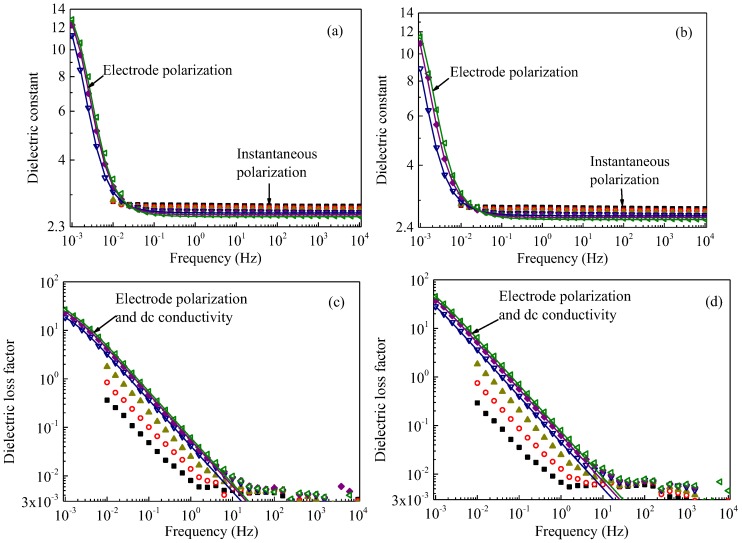
Dielectric constant *ε*_r_′ and dielectric loss factor *ε*_r_" each as a function of frequency, measured at various temperatures. Dielectric constant *ε*_r_′: (**a**) irradiated at 145 °C for 900 h and (**b**) irradiated at 145 °C for 1500 h. Dielectric loss factor *ε*_r_": (**c**) irradiated at 145 °C for 900 h and (**d**) irradiated at 145 °C for 1500 h. Symbols and solid curves represent the data obtained experimentally and numerically, respectively. The measurements were conducted at 100 °C (■), 120 °C (◯), 140 °C (▲), 160 °C (▽), 180 °C (♦), and 200 °C (◁).

**Figure 4:**
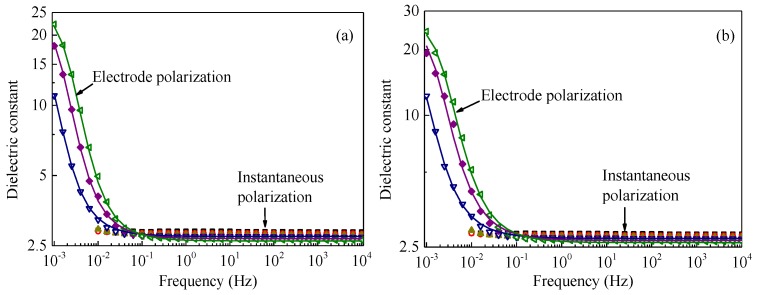

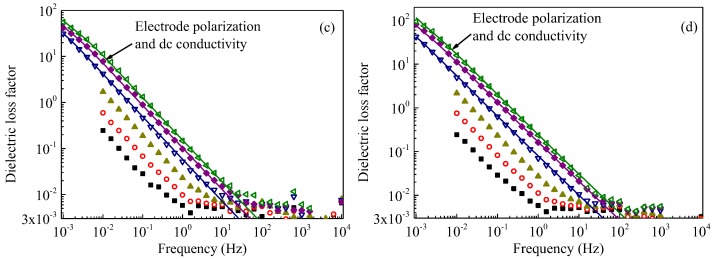
Dielectric constant *ε*_r_′ and dielectric loss factor *ε*_r_" each as a function of frequency, measured at various temperatures. Dielectric constant *ε*_r_′: (**a**) irradiated at 185 °C for 900 h and (**b**) irradiated at 185 °C for 1500 h. Dielectric loss *ε*_r_": (**c**) irradiated at 185 °C for 900 h and (**d**) irradiated at 185 °C for 1500 h. Symbols and solid curves represent the data obtained experimentally and numerically, respectively. The measurements were conducted at 100 °C (■), 120 °C (◯), 140 °C (▲), 160 °C (▽), 180 °C (♦), and 200 °C (◁).

Symbols with various colors and shapes in Figure 1, Figure 3 and Figure 4 show *ε*_r_*′* and *ε*_r_*′′* as a function of frequency obtained for all the five samples at temperatures from 100 to 200 °C. Solid curves are drawn to show simulation results obtained at temperatures of 140 °C and above for non-aged samples as shown in Figure 1 and at temperatures of 160 °C and above for aged samples as shown in Figure 3 and Figure 4. The simulation results are in good agreement with experiments for the pristine and all the gamma-irradiated samples, except for *ε*_r_*′′* at very high frequencies above 10 Hz in Figure 1, Figure 3 and Figure 4. The discrepancy of *ε*_r_*′′* between the values obtained by the simulation and experiments could be attributable to other relaxation processes and/or the inaccuracy of measurements.

**Figure 5:**
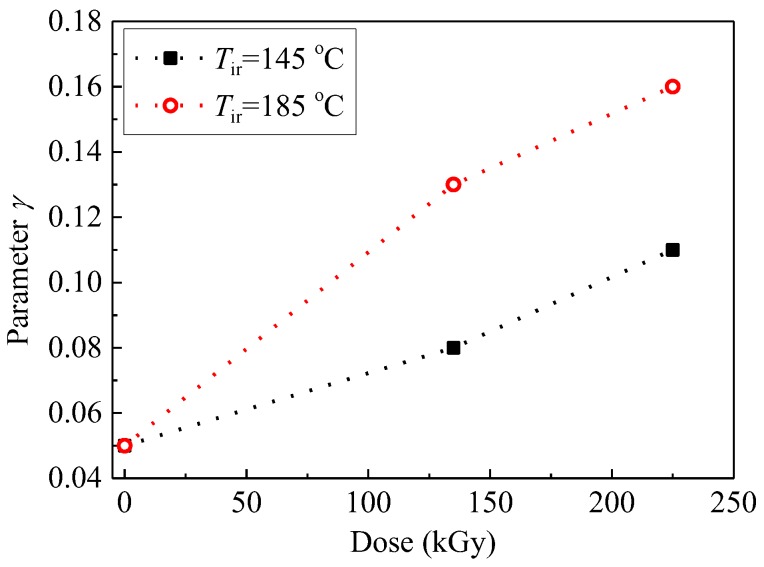
Parameter *γ* as a function of dose irradiated at temperatures *T*_ir_ of 145 and 185 °C.

From the above-mentioned simulations conducted for all the five samples, the values of parameters appearing in Equation (5) are all known. The parameter *γ* changes as shown in Figure 5, depending on the temperature and the duration of gamma irradiation. If *γ* is zero, the relaxation is a Debye-type relaxation, and it deviates more from the Debye type if *γ* becomes larger. As shown in Figure 5, the sample that was presumably degraded more has a big value of *γ*. This seems to be reasonable. For other parameters, such as *ε*_r∞_, *n*, *D*, and *σ*_DC_, and the effects of dose and temperature on them, will be discussed in Section 4.

## 4. Discussion

### 4.1. Influence of Degradation on the Dielectric Constant at High Frequencies

**Figure 6:**
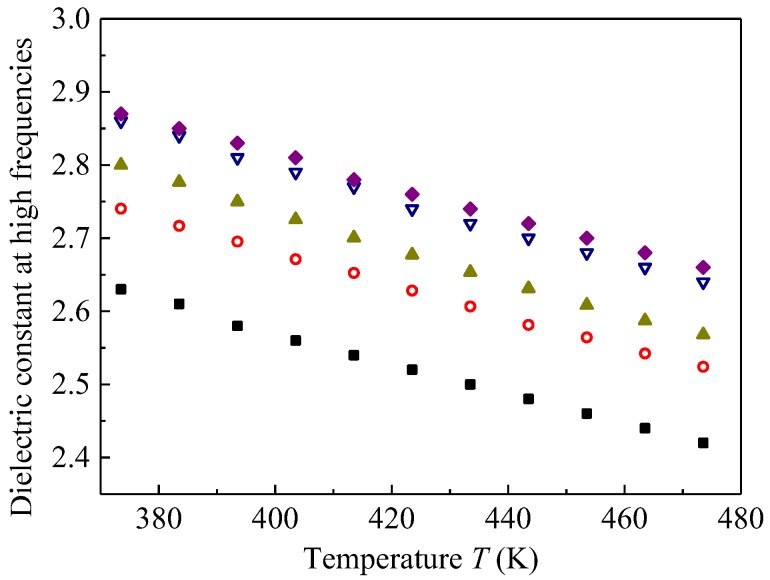
Dielectric constant at high frequencies above 10^3^ Hz *ε*_r*∞*_ as a function of measurement temperature *T*. ■: Pristine, ◯: irradiated at 145 °C for 900 h, ▲: irradiated at 145 °C for 1500 h, ▽: irradiated at 185 °C for 900 h, ♦: irradiated at 185 °C for 1500 h.

Figure 6 shows the effect of gamma irradiation to various doses given at two different temperatures (*T*_ir_) of 145 and 185 °C on the dielectric constant at the high frequency limit *ε*_r∞_ as a function of the measurement temperature, calculated by Equation (5). It is clear that *ε*_r∞_ increases with an increase in irradiation time *t*_ir_ for the ageing at both 145 and 185 °C, which may be caused by the increase in polarizability or density of polarizable substances. The relation between *ε*_r__∞_ and the polarizability is given by the following Clausius-Mossotti equation [22]:

(6) $$\frac{\varepsilon_{r\infty }-1}{\varepsilon_{r\infty }+2}=\frac{\rho N_{A}}{3\varepsilon_{0}M}\left(\alpha_{E}+\alpha_{A}\right)$$

Here, *α*_E_ is the electronic polarizability and *α*_A_ is the atomic polarizability, both in F·m^2^, while *ρ* is the density of the dielectric material in kg·m^−3^, *M* is the molecular weight in kg·mol^−1^, and *N*_A_ is the Avogadro constant in mol^−1^.

In our preceding research, chemiluminescence was measured for the present pristine and aged SiR samples [8]. It was found that the intensity of chemiluminescence becomes weak if the sample was aged concurrently by heat and gamma radiation. Oxidative reaction is detected as chemiluminescence and its intensity is proportional to the concentration of reactants [6,8]. Therefore, the decrease in chemiluminescence intensity with the increase in irradiation time means that the reactants were consumed during ageing [8]. Therefore, oxidative products, such as oxidation of side methyl groups, may have been formed in the degraded samples [6,8]. In addition, chain scission was induced by the concurrent ageing, leaving chain segments behind [9]. Oxidative products and chain segments may result in a change in atomic polarizability or skeletal vibrations in polymeric molecules, which should lead to an increase in atomic polarizability *α*_A_. Therefore, *ε*_r∞_ goes up with the gamma irradiation.

As shown in Figure 6, *ε*_r∞_ decreases monotonically with an increase in measurement temperature, showing a negative constant derivative with respect to temperature d*ε*_r_*_∞_*/d*T*. This is attributable to thermal expansion or the decrease in the number density of dipoles [12,23]. Equation (6) was used to analyze the temperature dependence of *ε_r_*_∞_.

**Figure 7:**
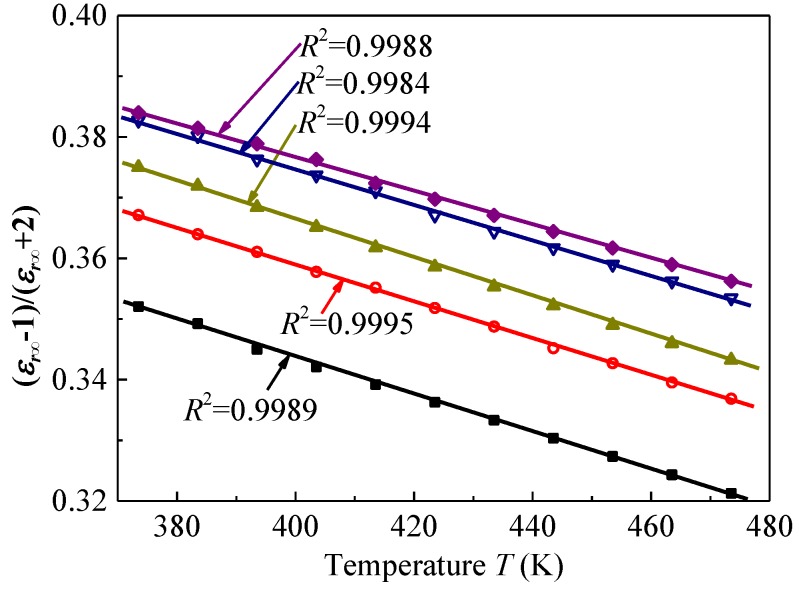
(*ε*_r*∞*_ − 1)/(*ε*_r*∞*_ + 2) as a function of temperature *T*. Symbols and solid curves represent the data obtained experimentally and numerically, respectively. *R*^2^ represents the goodness of fit. ■: Pristine, ◯: irradiated at 145 °C for 900 h, ▲: irradiated at 145 °C for 1500 h, ▽: irradiated at 185 °C for 900 h, ♦: irradiated at 185 °C for 1500 h.

Figure 7 shows (*ε*_r_*_∞_* − 1)/(*ε*_r_*_∞_* + 2) as a function of measurement temperature. It decreases linearly with the increase in temperature, which explains that the negative value of *dε*_r_*_∞_*/*dT* is caused by thermal expansion [12,23]. Therefore, the temperature-dependent *ε*_r_*_∞_* at temperatures from 100 to 200 °C can be used to investigate the influence of gamma irradiation on thermal expansion in SiR, which should be related to the concentrations of crosslinks and chain scissions. If the thermal expansion behavior is altered by the gamma irradiation, we can recognize that fact by drawing a figure like Figure 7, which should provide useful information on the degradation mechanism of SiR. The volume thermal expansion coefficient *β* was estimated by the Clausius-Mossotti equation. This method was already used to calculate *β* of EPDM [12], low-density polyethylene, poly(methyl pentene), and syndiotactic polystyrene [23] and found that the values of *β* obtained are in good agreement with those obtained by other methods, such as thermomechanical analysis and laser interference.

We assume that the density of SiR *ρ* is equal to *ρ*_0_ at *T*_0_ and has the following linear relation with temperature:

(7) $$\rho =\rho_{0}\left[1-\beta \left(T-T_{0}\right)\right]$$

We can calculate *d*[(*ε_r__∞_* − 1)/(*ε_r__∞_* + 2)]/*dT* by substituting Equation (7) into Equation(6):

(8) $$\frac{d}{dT}\left(\frac{\varepsilon_{r\infty }-1}{\varepsilon_{r\infty }+2}\right)=-\frac{\beta \rho_{0}N_{A}\left(\alpha_{E}+\alpha_{A}\right)}{3\varepsilon_{0}M}$$

We can obtain the following equation by replacing *ρ*_0_*N*_A_(*α*_E_+*α*_A_)/3*ε*_0_*M* on the right side in Equation (6) with Equations (7) and (8):

(9) $$\frac{\varepsilon_{r\infty }-1}{\varepsilon_{r\infty }+2}=T\frac{d}{dT}\left(\frac{\varepsilon_{r\infty }-1}{\varepsilon_{r\infty }+2}\right)-\left(\frac{1}{\beta }+T_{0}\right)\frac{d}{dT}\left(\frac{\varepsilon_{r\infty }-1}{\varepsilon_{r\infty }+2}\right)$$

From the values of (*ε*_r_*_∞_* − 1)/(*ε*_r_*_∞_* + 2) experimentally obtained as a function of *T*, we can calculate the slope *d*[(*ε*_r_*_∞_* − 1)/(*ε*_r_*_∞_* + 2)]/*dT*, and the intercept −(1/*β* + *T*_0_)*d*[(*ε*_r_*_∞_* − 1)/(*ε*_r_*_∞_* + 2)]/*dT* of Equation (9).

Figure 7 shows that the temperature dependence of (*ε*_r_*_∞_* − 1)/(*ε*_r_*_∞_* + 2) can be fitted well by Equation (9) and the values of *R*^2^ (goodness of fit) are larger than 0.998. Then, we can calculate (1/*β* + *T*_0_) with the values of the slope and the intercept. It was found that the value of (1/*β* + *T*_0_) is always larger than 1.5 × 10^3^ K. Since *T*_0_ is much smaller than 1.5 × 10^3^ K, we can estimate the thermal expansion coefficient *β* of SiR. As a result, we found that *β* ranges from 5.7 × 10^−4^ to 6.6 × 10^−4^ K^−1^.

**Figure 8:**
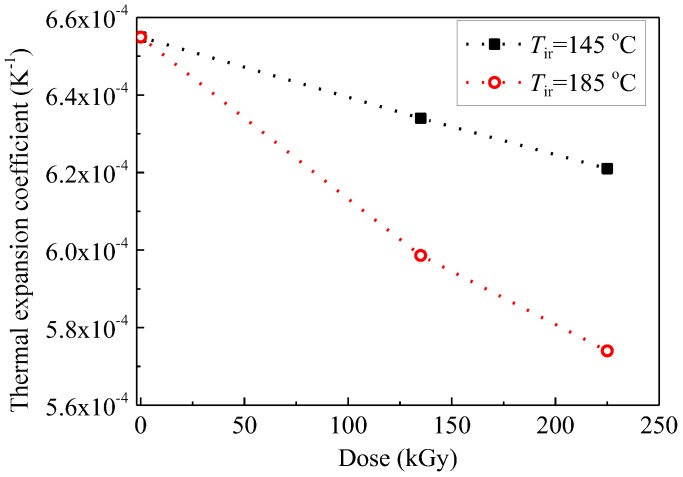
Thermal expansion coefficient *β* as a function of dose irradiated at temperatures *T*_ir_ of 145 and 185 °C.

**Figure 9:**
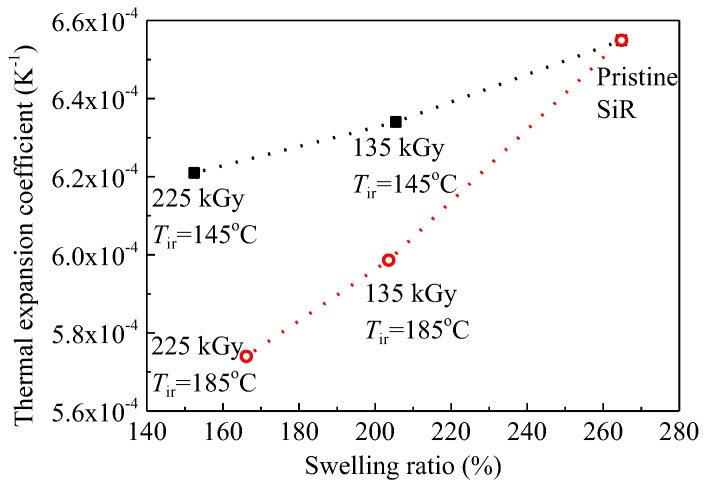
Thermal expansion coefficient *β* as a function of swelling ratio at *T*_ir_ of 145 and 185 °C.

Figure 8 shows that the thermal expansion coefficient *β* decreases with an increase in dose at *T*_ir_ of 145 and 185 °C. It may be caused by an increase in crosslinking degree or a decrease in swelling ratio induced by gamma irradiation. The swelling ratio was measured by the change in volume before and after immersing SiR samples into toluene for 24 h at room temperature [7]. The swelling ratios of SiR aged concurrently by heat and radiation decrease with the irradiation time, which means that the insoluble volume in toluene is increased by crosslinking. The relation between *β* and swelling ratio is shown in Figure 9. This indicates that the change in volume due to the increase in temperature becomes more difficult for the aged samples because crosslinking hinders the thermal expansion of molecular chains.

### 4.2. Influence of Degradation on Ion Concentration

**Figure 10:**
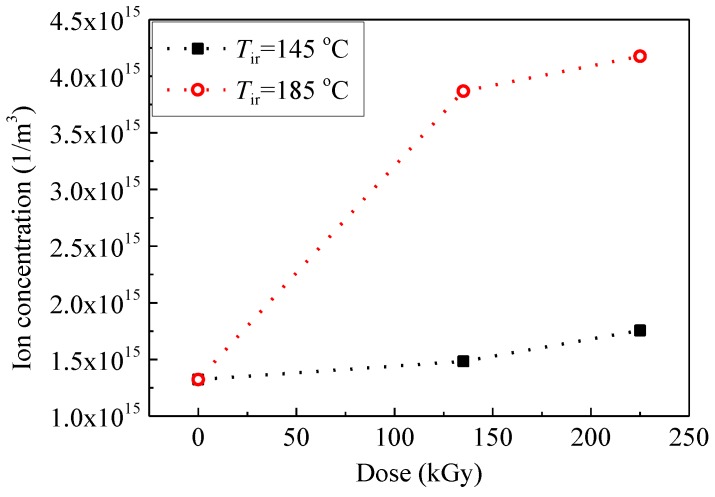
Ion concentration measured at 200 °C as a function of dose at *T*_ir_ of 145 and 185 °C.

**Figure 11:**
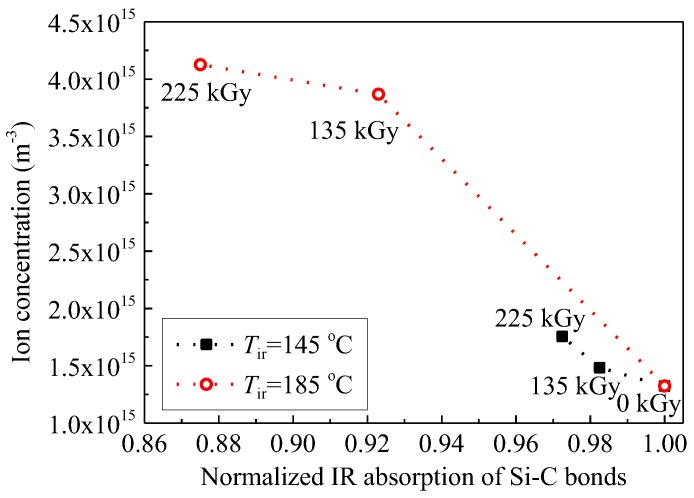
Ion concentration measured at 200 °C as a function of normalized absorption intensity of Si–C bonds observed at 785 cm^−1^ at *T*_ir_ of 145 and 185 °C.

Figure 10 shows that ion concentration *n* calculated by Equations (2) and (5) increases with an increase in dose. Segmental molecular chains in SiR can be excited and ionized by gamma irradiation. The energy of gamma rays, which is much higher than the bond energy and the ionization energy of molecules, will be transferred to extranuclear electrons. Excitation and ionization can thus be triggered. Meanwhile, ions which are generated inside the material would accumulate with an irradiation time. Fourier transform infrared absorption spectra of the pristine and aged SiR samples revealed that the absorption intensity of Si–C bonds between Si and CH_3_ at a wave number of about 785 cm^−1^ decreases with the increase in irradiation time [9]. This means that Si–C bonds were broken by the gamma irradiation. Figure 11 shows the relation between the ion concentration and the normalized absorption intensity of Si–C bonds. It is indicated that the generation of mobile ions is positively related with the number of chain scissions induced by gamma irradiation.

### 4.3. Influence of Degradation on Conductivity

**Figure 12:**
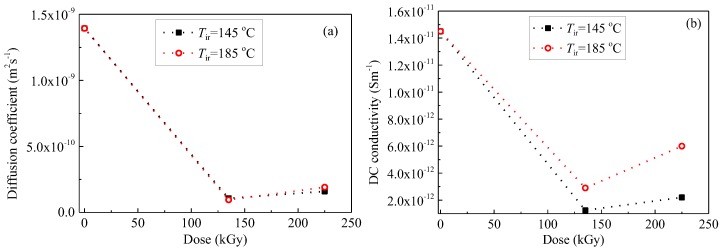
Diffusion coefficient *D* (**a**) and DC conductivity *σ_DC_* (**b**) as a function of dose calculated using experimental data obtained at 200 °C.

Figure 12 demonstrates the dose dependence of diffusion coefficient *D* and DC conductivity *σ*_DC_, measured at 200 °C. Both *D* and *σ*_DC_ decrease at first and then increase with the increase in dose. While *D* is the parameter that shows the easiness of ions, molecules, or atoms to change their positions in a medium, *σ*_DC_ is governed by the easiness of ions for changing the position in a medium under an applied electric field if the conduction current is due to ions. Namely, both *D* and *σ*_DC_ are directly related to the same phenomenon. Therefore, it is reasonable that the dose dependence is quite similar between *σ*_DC_ and *D*. It was found that Si–CH_3_ bonds in main molecular chains are broken by oxidation under the gamma irradiation and that Si–O–Si crosslinking points can be generated between two molecular chains [9]. The crosslinking reaction in SiR under gamma irradiation were confirmed by nuclear magnetic resonance (NMR). The NMR spectra shows that the intensity of Si atom bonded with three O atoms representing inter-molecular crosslinking points is increased after irradiation [7]. It means that the reactions of degradation and crosslinking occur simultaneously [2]. Many parameters such as irradiation time, dose rate, temperature, oxygen concentration, and so on would determine whether crosslinking or degradation occurs dominantly during the gamma irradiation. When crosslinking is dominant, a rigid, hard, and brittle structure will appear, which should suppress vibrations and motions of ions and molecules. However, when degradation dominates the reaction, molecular chains are broken, facilitating the motion of mobile ions and the decrease in molecular weight and mechanical strength. Figure 12 indicates that the reaction rate is higher for crosslinking than for chain scission at low doses of gamma irradiation, whereas the chain scission dominates the degradation process at high doses.

### 4.4. Influence of Degradation on Trap Distribution and Its Relation with Conductivity

**Figure 13:**
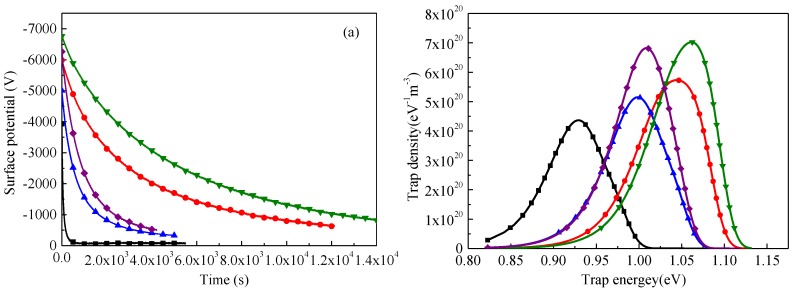
(**a**) Surface potentials of pristine and degraded SiR samples as a function of time, (**b**) trap distributions calculated by Equations (10) and (11). ■: Pristine SiR, ●: irradiated at 145 °C for 900 h, ▲: irradiated at 145 °C for 1500 h, ▼: irradiated at 185 °C for 900 h, ♦: irradiated at 185 °C for 1500 h.

As mentioned above, the conductivity of SiR is affected by crosslinking and scission of molecular chains, and these irradiation-induced reactions can change trap distributions in polymeric materials. Surface potential decay measurement thus is used to investigate the trap distributions of SiR before and after degradation. Figure 13a manifests that surface potential decays slower firstly and then faster with an increase in dose. The decay rate of surface potential becomes slower with an increase in ageing temperature when the irradiation time is fixed. In accordance with the surface potential decay theory [31,32,33], surface trap energy and trap density can be estimated by the following two equations by assuming that charge carriers are deposited in a layer with the thickness of *l*_s_ about 1 μm near the charging surface [33,34,35]:

(10) $$E_{T}=k_{B}T\ln\left(\upsilon_{\mathrm{ATE}}t\right)$$

(11) $$N_{T}=\frac{\varepsilon_{0}\varepsilon_{r}}{{edl}_{s}}t\frac{\partial \phi_{s}\left(t\right)}{\partial t}$$

where *E*_T_ is the energy of surface traps in eV, while *N*_T_ is the density of surface traps in eV^−1^·m^−3^, and *φ*_s_*(t)* is surface potential in V. Furthermore, *v*_ATE_ is the attempt-to-escape frequency in s^−1^,which can be expressed as *k*_B_*T*/*h*. Here, *h* is the Planck constant in J·s.

As demonstrated in Figure 13b, trap energy increases firstly and then decreases with an increase in dose at an ageing temperature of 145 or 185 °C. Irradiating SiR samples at higher temperature generates more traps inside. The trap densities of SiR samples degraded at 185 °C at the irradiation times of both 900 and 1500 h are larger than those of samples degraded at 145 °C. Chemical reactions of the formation of oxidative products, crosslinking, and chain scission change the energy and density of traps in polymers, which further results in the conductivity variation as demonstrated in Figure 12.

From the perspective of hopping conductivity theory, DC conductivity is exponentially proportional to trap energy, which is described by the following equation [12,22]:

(12) $$\sigma_{\mathrm{DC}}=\frac{q^{2}\lambda_{T}^{2}n_{0}\upsilon_{\mathrm{ATE}}}{6k_{B}T}\exp\left(-\frac{E_{T}}{k_{B}T}\right)$$

where *λ*_T_ is the hopping distance of charge carriers in m.

**Figure 14:**
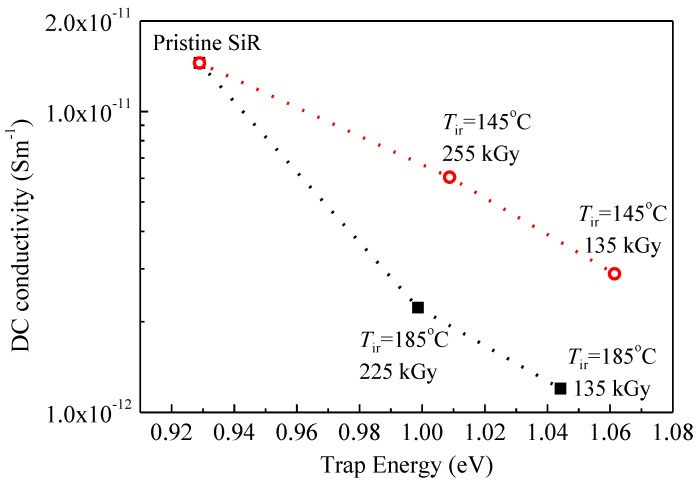
DC conductivity of SiR samples degraded by gamma irradiation at two different temperatures as a function of trap energy.

Figure 14 shows the relation between DC conductivity in Figure 12b and trap energy in Figure 13b. It is clear that DC conductivities of SiR samples degraded by gamma irradiation at temperatures of 145 and 185 °C decrease exponentially with an increase in trap energy, which obeys hopping conductivity Equation (12). As mentioned in Figure 13, a small amount of gamma irradiation can augment the energy of traps in SiR by triggering chain crosslinks inside. Accordingly, the hopping of charge carriers becomes more difficult and conductivity decreases. When the irradiation time is long enough, the reaction rate of chain scission becomes larger than that of crosslinking. Meanwhile, the energy of traps is reduced and charge carriers can hop easier in the material. Therefore, DC conductivity increases again at higher dose of gamma irradiation. In Figure 13b, it is also shown that the densities of traps in SiR samples degraded at 185 °C are higher than those degraded at 145 °C. Since the hopping distance equals to the reciprocal of trap density to the power of 1/3, namely, *λ*_T_ = 1/*N*_T_^1/3^, the hopping distance of charge carriers is smaller in SiR samples degraded at 185 °C. Consequently, the conductivities of SiR samples degraded at 185 °C are lower than those degraded at 145 °C accordingly to Equation (12).

It has been shown that the dielectric constant at high frequency limit *ε*_r∞_, thermal expansion coefficient calculated from temperature dependence of *ε*_r∞_, ion concentration calculated from electrode polarization at relatively low frequencies change monotonically with irradiation time. Their chemical origins were also analyzed. DC conductivity decreases firstly and then increases with an increase in irradiation time, which can be well explained by the variations of trap energy and density induced by gamma irradiation. It indicates that dielectric spectroscopy is a powerful tool to investigate the degradation mechanism and to monitor the condition of power cables used in nuclear power plants.

## 5. Conclusions

Complex permittivity spectra were measured for silicone rubber sheets aged concurrently by heat and gamma irradiation. The resultant changes in thermal expansion coefficient, ion concentration, diffusion coefficient, and induced DC conductivity were analyzed.

- The dielectric constant at high frequencies increases with an increase in irradiation time and ageing temperature. This indicates that oxidative products are formed during gamma irradiation.
- It is clearly shown that the thermal expansion coefficients can be calculated via the Clausius-Mossotti equation from the temperature dependence of the dielectric constant at high frequencies. The coefficient decreases with an increase in irradiation time and ageing temperature, indicating that the crosslinking reaction occurs dominantly during the ageing process, which is in good agreement with the results on the swelling ratio.
- The concentration of ions increases with the increase in irradiation time and ageing temperature. This is seemingly caused by the increase in bond break or chain scission, which is consistent with the decrease in infrared absorption intensity due to Si–C bonds.
- The diffusion coefficient and DC conductivity decrease at first and then increase with the increase in dose. From the viewpoint of the carrier hopping model, DC conductivity is determined by trap energy. It was found that trap energy calculated from the surface potential decay experiments can be used to interpret the variation of the DC conductivity of SiR with an increase in dose. This indicates that crosslinking is dominant at low doses and that chain scission becomes dominant at high doses.

## References

[1] Gonzalez-Perez G., Burillo G., Ogawa T., Avalos-Borja M. (2012). Grafting styrene and 2-vinylnaphthalene onto silicone rubber to improve radiation resistance. Polym. Degrad. Stab..

[2] Shimada A., Sugimoto M., Kudoh H., Tamura K., Seguchi T. (2014). Degradation mechanisms of silicone rubber (SiR) by accelerated ageing for cables of nuclear power plant. IEEE Trans. Dielectr. Electr. Insul..

[3] Roggero A., Dantras E., Paulmier T., Tonon C., Balcon N., Rejsek-Riba V., Dagras S., Payan D. (2015). Electrical behaviour of a silicone elastomer under simulated space environment. J. Phys. D.

[4] Verdu J. (2012). Oxidative Ageing of Polymers.

[5] Min D.M., Li S.T., Hirai N., Ohki Y. (2016). Modeling of Oxidation Process and Property Changes of Ethylene-Propylene-Diene Copolymer. IEEE Trans. Dielectr. Electr. Insul..

[6] Chaudhry A.N., Billingham N.C. (2001). Characterisation and oxidative degradation of a room-temperature vulcanised poly(dimethylsiloxane) rubber. Polym. Degrad. Stab..

[7] Ohki Y., Hanada S., Miyamoto M., Hirai N., Yang L.Q. Aging mechanism of silicone rubber by heat and gamma-rays. Proceedings of the IEEE Conference on Electrical Insulation and Dielectric Phenomena (CEIDP).

[8] Ikeno R., Hirai N., Ohki Y. Chemiluminescence characteristics of FR-EPDM and SiR aged by concurrently-given heat and radiation. Proceedings of the IEEE Conference on Electrical Insulation and Dielectric Phenomena (CEIDP).

[9] Hanada S., Odaka D., Yang L.Q., Hirai N., Ohki Y. Non-destructive diagnosis of degradation of silicone rubber by indenter modulus and scanning probe microscopy. Proceedings of the International Conference on Condition Monitoring and Diagnosis (CMD).

[10] Hanada S., Miyamoto M., Hirai N., Yang L., Ohki Y. (2017). Experimental investigation of the degradation mechanism of silicone rubber exposed to heat and gamma rays. High Volt..

[11] Verardi L., Fabiani D., Montanari G.C. (2014). Electrical aging markers for EPR-based low-voltage cable insulation wiring of nuclear power plants. Radiat. Phys. Chem..

[12] Min D.M., Li S.T., Hirai N., Ohki Y. (2016). Dielectric Spectroscopic Analysis of Degradation in Ethylene-Propylene-Diene Copolymer. IEEE Trans. Dielectr. Electr. Insul..

[13] Labouriau A., Cady C., Gill J., Stull J., Ortiz-Acosta D., Henderson K., Hartung V., Quintana A., Celina M. (2015). Gamma irradiation and oxidative degradation of a silica-filled silicone elastomer. Polym. Degrad. Stab..

[14] Seguchi T., Tamura K., Ohshima T., Shimada A., Kudoh H. (2011). Degradation mechanisms of cable insulation materials during radiation–thermal ageing in radiation environment. Radiat. Phys. Chem..

[15] Yamamoto T., Minakawa T. (2009). Japan Nuclear Energy Safety Organization Final Report of the Project of Assessment of Cable Ageing for Nuclear Power Plants.

[16] Simmons K.L., Pardini A.F., Fifield L.S., Tedeschi J.R., Westman M.P., Jones A.M., Ramuhalli P. (2013). Determining Remaining Useful Life of Aging Cables in Nucleat Power Plants—Interim Study FY13.

[17] Linde E., Verardi L., Fabiani D., Gedde U.W. (2015). Dielectric spectroscopy as a condition monitoring technique for cable insulation based on crosslinked polyethylene. Polym. Test..

[18] Kortschot R.J., Philipse A.P., Erné B.H. (2014). Debye length dependence of the anomalous dynamics of ionic double layers in a parallel plate capacitor. J. Phys. Chem. C.

[19] Klein R.J., Zhang S.H., Dou S.C., Jones B.H., Colby R.H., Runt J. (2006). Modeling electrode polarization in dielectric spectroscopy: Ion mobility and mobile ion concentration of single-ion polymer electrolytes. J. Chem. Phys..

[20] Masuzaki Y., Ohki Y., Kozako M. Comparison of dielectric properties among polydicyclopentadiene resin, epoxy resin and their composites with microsized SiO_2_ Fillers. Proceedings of the IEEE International Symposium on Electrical Insulating Materials (ISEIM).

[21] Li X.T., Masuzaki Y., Tian F.Q., Ohki Y. (2015). Space charge formation and charge transport in epoxy resin at varied temperatures. IEEJ Trans. Fundam. Mater..

[22] Kao K.C. (2004). Dielectric Phenomena in Solids.

[23] Hasegawa Y., Takihana J., Ohki Y. (2014). Estimation of thermal expansion coefficients of polymeric insulating films from temperature dependence of dielectric permittivity. Jpn. J. Appl. Phys..

[24] Sidebottom D.L. (2009). Colloquium: Understanding ion motion in disordered solids from impedance spectroscopy scaling. Rev. Mod. Phys..

[25] Ishai P.B., Talary M.S., Caduff A., Levy E., Feldman Y. (2013). Electrode polarization in dielectric measurements: A review. Meas. Sci. Technol..

[26] Diaham S., Locatelli M.L. (2012). Concentration and mobility of charge carriers in thin polymers at high temperature determined by electrode polarization modeling. J. Appl. Phys..

[27] Tian F.Q., Ohki Y. (2014). Charge transport and electrode polarization in epoxy resin at high temperatures. J. Phys. D.

[28] Macdonald J.R. (1953). Theory of ac Space-Charge Polarization Effects in Photoconductors, Semiconductors, and Electrolytes. Phys. Rev..

[29] Cole K.S., Cole R.H. (1941). Dispersion and absorption in dielectrics I. Alternating current characteristics. J. Chem. Phys..

[30] Cole K.S., Cole R.H. (1942). Dispersion and absorption in dielectrics II. Direct current characteristics. J. Chem. Phys..

[31] Simmons J.G., Tam M.C. (1973). Theory of isothermal currents and the direct determination of trap parameters in semiconductors and insulators containing arbitrary trap distributions. Phys. Rev. B.

[32] Simmons J.G., Taylor G.W., Tam M.C. (1973). Thermally stimulated currents in semiconductors and insulators having arbitrary trap distributions. Phys. Rev. B.

[33] Li J.Y., Zhou F.S., Min D.M., Li S.T., Xia R. (2015). The Energy Distribution of Trapped Charges in Polymers Based on Isothermal Surface Potential Decay Model. IEEE Trans. Dielectr. Electr. Insul..

[34] Huang Y., Min D.M., Li S.T., Li Z., Xie D.R., Wang X., Lin S.J. (2017). Surface flashover performance of epoxy resin microcomposites improved by electron beam irradiation. Appl. Surf. Sci..

[35] Wintle H.J. (1971). Decay of Surface Electric Charge in Insulators. Jpn. J. Appl. Phys..

